# Routine Hair Testing Unmasks Hidden Synthetic Cannabinoid Use in Forensic Psychiatric Patients: A 10-Year Comparative Study in Two Bavarian Clinics

**DOI:** 10.3390/brainsci15111240

**Published:** 2025-11-19

**Authors:** Michael Fritz, Hannah Funk, Felipe Montiel, Judith Streb, Manuela Dudeck

**Affiliations:** 1 Department of Forensic Psychiatry and Psychotherapy, Ulm University, 89312 Guenzburg, Germany; 2 Clinic for Forensic Psychiatry and Psychotherapy, District Hospital Kaufbeuren, 87600 Kaufbeuren, Germany; felipe.montiel@bkh-kaufbeuren.de

**Keywords:** synthetic cannabinoids, forensic psychiatry, abstinence monitoring, substance use disorders, violence

## Abstract

Background: Germany provides a worldwide almost unique legal framework for offenders with substance use disorders through § 64 of the German Criminal Code, mandating a two-year multimodal therapy including an in-house clinical treatment period followed by a reintegration phase with gradually reduced supervision. During this phase, lapses are often concealed, with synthetic cannabinoids (SCs) serving as a potential tool due to limited detection in routine screenings and heterogeneous monitoring practices across forensic psychiatric clinics. Methods: This study compared two forensic hospitals, Guenzburg and Kaufbeuren, over a ten-year period, from 2014 to 2024, to evaluate monitoring strategies. While Kaufbeuren applied a case-dependent testing approach, including unannounced urine screenings, Guenzburg introduced a stricter regime in 2019, combining mandatory hair analysis three months into reintegration with unannounced broad-spectrum screenings including SCs. Results: Among the 527 patients included in this study, significantly more tests were conducted in Guenzburg after 2022. The different approach between hair vs. urine analysis produced a seven-fold higher detection rate of SC use compared to Kaufbeuren. Across both clinics, however, SC-positive patients shared similar features. They were younger at first conviction, more frequently under substitution treatment, and more likely to have committed violent offenses. A history of violence quadrupled SC-positive odds, while time since leave as such increased odds by 0.1% per day. Conclusions: In conclusion, these results underscore the effectiveness of standardized long-term SC monitoring using hair analysis and the predictive role of a history of violence in the context of SC-relapse.

## 1. Introduction

Forensic psychiatry clinics worldwide primarily treat individuals who have committed crimes in connection with severe mental disorders, such as schizophrenia spectrum disorders or personality disorders [1]. While institutional structures are largely similar across countries, standards of care and treatment procedures differ [2]. Germany represents a notable exception, as it is among the few countries that also mandate forensic treatment for offenders with substance use disorders (SUDs; [3]). According to § 64 of the German Criminal Code, offenders may be sentenced to a two-year forensic psychiatric therapy program if their crimes are attributable to a SUD, including drug trafficking, acquisitive crimes committed to obtain drugs, or violent offenses committed under the influence of substances [4].

Alcohol use, for instance, is highly relevant in this context. The criminogenic effects of alcohol and the high prevalence of alcohol use disorders among incarcerated individuals are well documented across both sexes [5,6]. However, elevated levels of criminal behavior have also been observed among users of other substances, such as opioids [7] and cocaine [8]. More broadly, substance use has been associated with increased aggression, as demonstrated in a recent meta-analysis [9], necessitating the German approach. The legal objective of the § 64 treatment is the integration or reintegration of offenders into society, personal development, and the prevention of reoffending [10]. A major challenge, however, is the compulsory nature of treatment, which often results in initially low therapeutic motivation and socially desirable, rather than genuinely change-oriented, behavior [11,12].

Treatment within § 64 institutions is multimodal, comprising individual and group psychotherapy, occupational and work therapy, sport and music therapy, and medical care, including substitution treatment [4]. The initial phase of therapy takes place under secure inpatient conditions, but restrictions are gradually reduced to facilitate social reintegration. In this phase, patients typically live independently and work externally, while maintaining contact with the clinic through weekly to biweekly counseling sessions and periodic abstinence monitoring—a process which is best described as trial leave. At this stage, sustained abstinence becomes especially critical, as therapeutic success and behavioral change are tested in real-world contexts. Relapse risk is influenced by factors such as emotional distress, waning motivation, social pressure, or recklessness [13], while fear of legal consequences, shame, and the deliberate concealment of consumption further complicate relapse detection [14,15].

A particular challenge arises from the detection of synthetic cannabinoids (SCs), which are difficult to identify with routine urine screening [15,16]. Although the overall societal prevalence of SC use appears low, detailed epidemiological investigations remain scarce, and reported rates vary considerably across populations. In the United States, a national survey found an increase in SC use from 0.17% in 2021 to 0.26% in 2023 [17]. In contrast, a German study reported a 12-month prevalence of 1.3% among individuals aged 18 to 64 years in 2021 [18]. However, markedly higher rates have been observed in specific subgroups. For example, Craft et al. [19] found that 45% of English prison inmates self-reported SC use between 2015 and 2022, highlighting its elevated prevalence among individuals with antisocial personality traits. These inmates also reported greater levels of psychological distress. Similarly, Hobbs et al. [20] documented a rapid increase in SC use among patients in South London between 2013 and 2017, with consumption patterns associated with homelessness and increased hospital inpatient days.

Due to the population-specific heightened prevalence and the difficulty of detecting SCs with routine screening methods, their use may undermine contingency management and other addiction treatments if relapses go undetected. Despite this, there are no standardized national procedures for abstinence monitoring, and practices vary considerably across German forensic psychiatric clinics. While urine tests are common throughout Germany, long-term hair analyses are used less frequently due to higher costs. As a result, relapses involving SCs may remain unnoticed, jeopardizing the therapeutic aim of promoting lasting abstinence and preventing recidivism.

The present study addresses this gap by comparing two forensic psychiatric clinics in Bavaria with different approaches to drug monitoring during the social reintegration phase. Specifically, we examine whether differences exist between the district hospital Kaufbeuren, where urine tests for SCs are only performed in cases of suspicion, and Guenzburg, where routine hair analyses for SCs are commonly performed and are conducted irrespective of suspicion. We investigate (1) differences between Kaufbeuren and Guenzburg with regard to the frequency of SC-positive cases, (2) the types of SCs involved in relapses, (3) temporal variations between 2014 and 2024 in relapse frequency, and (4) the influence of sociodemographic, psychiatric, or criminal characteristics on SC relapse.

## 2. Methods

### 2.1. Sample

A total of 540 patients who were granted trial leave from the forensic psychiatric hospitals in Kaufbeuren (*n* = 423) and Guenzburg (*n* = 117) between 2014 and 2024 were initially identified. Medical records were unavailable for 13 patients (2%; Kaufbeuren: *n* = 6, Guenzburg: *n* = 7), leading to their exclusion. The final analytical sample therefore comprised 527 patients (Kaufbeuren: *n* = 417; Guenzburg: *n* = 110).

### 2.2. Procedure

Data from the district hospitals Kaufbeuren and Guenzburg, Germany, were collected using a standardized survey form during a fully anonymized file review, without direct patient contact. Information was extracted from medical records, including primary diagnosis (ICD-10), comorbid personality disorder, number of prior convictions, type of index offense, previous offenses, age at first conviction, and substance relapse during leave involving synthetic cannabinoids (as defined by FTC laboratory guidelines). It is important to note that this study does not distinguish between a “lapse” and a “relapse.” Its focus lies on the necessity of reliable abstinence testing, referring to any positive laboratory result as a “relapse.” Whether such a positive test represents an isolated incident (i.e., a “lapse”) or a return to substance use behaviors consistent with Substance Use Disorders (i.e., a “relapse”), as defined in the literature [21], was not assessed in this work.

All procedures were conducted in accordance with the ethical standards of national and institutional research committees and the principles of the Declaration of Helsinki (1975, revised 2013). The requirement for informed consent was waived due to the retrospective design and the exclusive use of anonymized data. The associated project number of the Ethics Committee of Ulm University is 466/22 (date 16 February 2023).

### 2.3. Statistical Analysis

To address the first research question, the number of positive and negative test results for synthetic cannabinoids was reported separately for each hospital. Group differences were assessed using the Chi-square test or Fisher’s exact test when expected cell counts were low. A binary logistic regression was then conducted with relapse (1 = positive, 0 = negative) as the dependent variable and hospital (0 = Guenzburg, 1 = Kaufbeuren) and time since leave (days) as independent variables. Results are reported as odds ratios (ORs) with 95% confidence intervals (CIs).

For the second and third research questions, absolute and relative frequencies were calculated and compared using Fisher’s exact test. To address the fourth research question, bivariate analyses were first conducted to compare patients with and without relapse. Categorical variables were summarized with counts and percentages and compared using Chi-square tests, while continuous variables were summarized with means and standard deviations and compared using independent-samples *t*-tests. The Mann–Whitney U test was applied to non-normally distributed variables.

Variables showing significant group differences were subsequently included in a binary logistic regression model, with relapse as the dependent variable (1 = positive, 0 = negative). All covariates, including time since leave, were entered simultaneously. Results are reported as ORs with 95% CIs. Statistical significance was defined as *p* < 0.05. All analyses were performed using SPSS version 29 (IBM, Armonk, NY, USA).

## 3. Results

Sociodemographic, clinical, and forensic characteristics of the two samples are presented in Table 1. Patients from the District Hospital in Guenzburg were more frequently diagnosed with F12 and less frequently with F19. They more often had a comorbid personality disorder, were more frequently undergoing substitution treatment, and, on average, had longer periods of hospitalization compared to patients from the District Hospital in Kaufbeuren.

Next, we examined whether the number of positive tests for synthetic cannabinoids differed between Kaufbeuren, where testing was conducted only upon suspicion primarily using urine analysis, and Guenzburg, where routine testing was performed using hair analysis.

**Figure 1:**
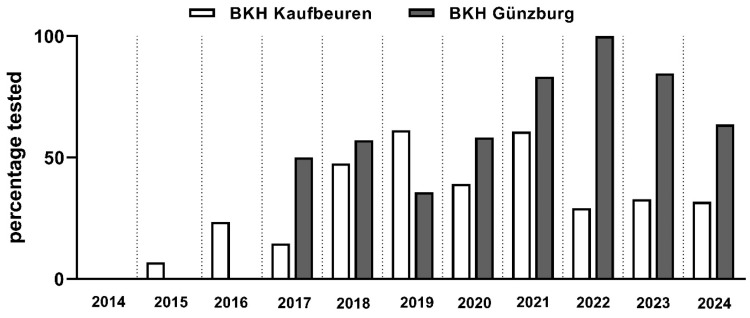
Proportion of tested patients among those on leave over time, shown separately for the study sites in Guenzburg and Kaufbeuren.

Accordingly, Figure 1 demonstrates that, beginning in 2022, the District Hospital in Guenzburg conducted significantly more tests for synthetic cannabinoids relative to the number of patients on leave compared to the District Hospital in Kaufbeuren (2022: χ^2^(1) = 16.34, *p* < 0.001, Cramer-*V* = 0.505; 2023: χ^2^(1) = 14.16, *p* < 0.001, Cramer-*V* = 0.426; 2024: χ^2^(1) = 9.89, *p* = 0.002, Cramer-*V* = 0.584), even though the District Hospital in Kaufbeuren started testing for SCs earlier. Across all years, a significantly higher proportion of patients on leave were tested at the District Hospital in Guenzburg (61.8%; 68/110) than at the District Hospital in Kaufbeuren (38.6%; 161/417), χ^2^(1) = 19.08, *p* < 0.001, Cramer-*V* = 0.190.

As shown in Table 2, 15 of the 68 tested patients in Guenzburg were positive for synthetic cannabinoids, resulting in a positivity rate of 22.1%. In Kaufbeuren, 6 of 161 tested patients were positive, corresponding to a rate of 3.7%. These rates differed significantly (χ^2^(1) = 19.29, *p* < 0.001, Cramer-*V* = 0.290). Based on the total number of patients (110 in Guenzburg and 417 in Kaufbeuren), the overall prevalence of positive cases was 13.6% in Guenzburg and 1.4% in Kaufbeuren.

In the next step, a binary logistic regression analysis was conducted. The results of this analysis aimed at predicting relapse with synthetic cannabinoids (relapse = 1, no relapse = 0) based on the predictors *site of data collection* (Guenzburg = 1, Kaufbeuren = 0) and *duration from leave to test* (in days) are presented in Table 3.

The omnibus test of model coefficients was significant, χ^2^(2) = 16.90, *p* < 0.001, indicating that the model distinguished between patients with and without relapse. The Hosmer–Lemeshow goodness-of-fit test suggested an adequate model fit, χ^2^(8) = 19.94, *p* = 0.10. The model explained approximately 15.9% of the variance in relapse (Nagelkerke’s *R*^2^ = 0.159). The odds ratio for the site of data collection was significant, indicating that patients in Guenzburg were seven times more likely to test positive for synthetic cannabinoids compared to those in Kaufbeuren.

Although SCs share the same pharmacological target as Δ^9^-tetrahydrocannabinol (Δ^9^-THC)—the cannabinoid type 1 (CB_1_) receptor—there are multiple classes of SCs, such as ada-mantoylindole, benzoylindoles, cyclohexylphenols, classical diben-zopyrans, indazole-based naphthoylindoles and WIN 55,212-2,naphthoylpyrroles, naphthylmethylindoles, naphthylmethylin-denes, phenylacetylindoles, quinolinyl ester, etc. [22,23]. Preclinical studies have confirmed reinforcing effects for some compounds within the A, C, and P classes [24,25], whereas other SCs, such as JWH-18, appear aversive in animal models [26]. Interestingly, the most frequently detected substance classes in relapse events in this study were H and M (see Table 4), despite evidence that compounds like HU-210 induce conditioned place preference in rats only after extensive pretreatment prior to behavioral training [27].

After identifying the most commonly used classes of SCs between 2014 and 2024, we next examined whether the number of relapses changed over time. The frequency of positive tests for SCs remained relatively stable across the study period (Fisher’s exact test = 15.926, *p* = 0.060, Cramer-*V* = 0.295; Table 5).

To address the fourth research question—whether any sociodemographic, psychiatric, or criminal characteristics were associated with positive SC test results—we conducted correlation analyses. Each recorded variable was first examined individually to assess potential differences between patients who relapsed and those who did not. As shown in Table 6, patients who tested positive for SCs were more frequently receiving substitution treatment, were younger at the time of their first conviction, and were more likely to have committed a violent offense than those who tested negative.

In the final step, we examined whether any of the variables that correlated with SC relapses also served as predictors. Variables that differed between patients with positive and negative test results, as well as time since leave, were included in a binary logistic regression model, with the dependent variable being coded as 1 = relapse with synthetic cannabinoids and 0 = no relapse.

The omnibus test of model coefficients was significant (χ^2^(4) = 12.064, *p* = 0.017), indicating that the model reliably distinguished between patients with and without SC relapses. The Hosmer–Lemeshow goodness-of-fit test showed no evidence of poor model fit (χ^2^(8) = 8.587, *p* = 0.378), and Nagelkerke’s *R*^2^ was 0.115. Table 7 presents the odds ratios of the predictors, representing the change in odds of a positive test associated with a one-unit increase in each predictor, controlling for the others. A history of violent offenses increased the odds of testing positive for synthetic cannabinoids by approximately fourfold, and time since leave increased the odds by 0.1% per day. Patients on substitution treatment showed 3.5-fold increased odds of SC use, approaching statistical significance (*p* = 0.067), possibly reflecting polysubstance use patterns. Age at first conviction, however, did not reach significance.

## 4. Discussion

The present study aimed to examine potential differences between the forensic psychiatric hospitals in Kaufbeuren and Guenzburg (Germany) in the detection of SCs between 2015 and 2025. In Kaufbeuren, testing was performed only in cases of clinical suspicion and was primarily based on urine analysis, whereas in Günzburg, testing was conducted routinely, irrespective of suspicion, and predominantly relied on hair analysis. Furthermore, the study investigated whether the frequency of SC relapses changed over the past decade and whether specific classes of synthetic cannabinoids predominated among positive test results. Finally, it explored whether sociodemographic, psychiatric, or criminal characteristics were associated with SC relapse and whether these factors could serve as predictors by increasing the odds of relapse.

While the District Hospital of Guenzburg tested a larger proportion of its patients, including some potentially lower-risk individuals, the positivity rate among those tested was substantially higher. This indicates that the difference cannot be attributed to testing frequency alone. The observed discrepancy in positivity rates is likely due to methodological differences in testing strategies. Guenzburg employed hair analysis, which provides a longer detection window and higher sensitivity for past substance use, whereas Kaufbeuren relied primarily on urine testing, which detects only recent consumption of up to three days [23]. Hair samples, on the other hand, offer several advantages over other biological matrices in forensic analysis, the most important being the chemical stability of target compounds and a longer detection window [28]. Moreover, the limits of detection differ by approximately one order of magnitude in favor of hair analysis—less than 1 ng/mL in urine [29] compared to 0.5–5 pg/mg in hair [28]. Consequently, even single lapses are more easily detected in hair than in urine. This methodological distinction likely accounts for the considerably higher positivity rate in Guenzburg despite its broader testing coverage. Extrapolating from these findings, approximately 30 additional patients (i.e., 36 instead of 6) at the District Hospital in Kaufbeuren might have been identified as relapsed had hair testing been implemented systematically.

Although no significant temporal changes in the number of SC-positive tests were observed across the study period, several predictors for relapse risk were identified. A history of violent offending increased the odds of an SC-related relapse fourfold, while the time since discharge from inpatient treatment increased the odds of relapsing by approximately 0.1% per day.

The association between a history of violent offenses and an increased likelihood of relapse with SCs is striking and warrants further investigation. This finding is particularly intriguing given that the anti-aggressive effects of Δ^9^-THC through CB_1_ receptor interactions have been demonstrated preclinically across species since the late 1970s [30,31] and have also been observed in humans [32].

In contrast to Δ^9^-THC, however, SCs exhibit distinct pharmacological profiles. Although the group encompasses a wide range of compounds, most SCs act as full CB_1_ receptor agonists with considerably higher receptor affinity and intrinsic activity than Δ^9^-THC [33]. Moreover, they lack naturally occurring cannabinoids such as cannabidiol and cannabivarin, both of which exert antipsychotic and modulatory effects on Δ^9^-THC-induced psychoactivity [34]. Consequently, SC consumption is more likely to provoke agitation and irritability [35]. At the same time, little is known about the long-term consequences of chronic SC use. Evidence of self-mutilation leading to severe bodily harm, including amputations, suggests an alarming reduction in thresholds for (auto)-aggressive behavior [36]. Similarly, one of the few studies investigating the relationship between aggression and SC use found associations with sexual violence, weapon possession, and physical fights among students reporting lifetime SC use [37].

Hence, it is plausible that individuals with specific personality traits may be drawn to SCs as a means of reinforcing or accommodating their characteristic behavioral tendencies. Substantial evidence supports a high comorbidity between personality disorders and substance use disorders in general [38], particularly between antisocial personality disorder (ASPD) and alcohol use disorder [39]. Notably, between 60% and 80% of forensic psychiatric patients meet criteria for a personality disorder, with ASPD being the most prevalent, affecting approximately 38% [40,41].

An alternative explanation may also involve the manipulative and deceptive tendencies commonly observed in individuals with antisocial traits [42] (Giammarco et al., 2013). In this context, SCs might be intentionally chosen to alleviate craving associated with substance use disorders while minimizing the likelihood of detection, given the analytical challenges of identifying these substances.

Finally, although only a minority of individuals (7.2%) who use both natural cannabis and synthetic cannabinoids report preferring the latter [43], it cannot be ruled out that this preference serves a self-medication purpose. This seems plausible given that these users cited the more pronounced positive psychotropic effects of synthetic cannabinoids as their reason for preference. Such effects—typically described as a “heavy buzz” or an intense feeling of being “stoned” [44]—might be perceived as a means to alleviate aggressive tensions, although paradoxically they may instead exacerbate them.

## 5. Limitations

One limitation of this study lies in its actuarial design. Although medical records provide detailed and reliable information, they may also contain omissions or inaccuracies due to human error and were not originally compiled to address the specific research questions posed in this work. Furthermore, the absence of direct patient contact prevents insight into the underlying motives for choosing synthetic cannabinoids as a means of relapse. Consequently, the hypotheses formulated in the discussion require further empirical investigation with patient participation to be verified or refuted. Finally, the inability to distinguish between lapse and relapse, combined with the lack of information regarding individual motives, limits the strength of any conclusions about high-risk profiles.

## 6. Conclusions

In summary, the findings indicate that methodological differences in testing strategies substantially influence the detection of synthetic cannabinoid use in forensic psychiatric settings. Beyond these procedural factors, a history of violent offending emerged as a strong predictor of relapse, underscoring the interplay between behavioral predispositions and substance use patterns. These results highlight the need for systematic, sensitive screening approaches and for targeted preventive interventions addressing high-risk individuals in forensic psychiatric populations.

## Figures and Tables

**Table 1:** Sociodemographic, clinical, and forensic characteristics of the sample, separately for patients from the District Hospitals in Guenzburg and Kaufbeuren.

	Guenzburg (*n* = 110) *M* (*SD*)/*n* (%)	Kaufbeuren (*n* = 417) *M* (*SD*)/*n* (%)	Statistics
Sex			FET = 11.438, *p* = 0.009, Cramer-*V* = 0.147
male	107 (97%)	417 (100%)	
female	3 (3%)	0	
Age at the beginning of leave (in years)	36.01 (8.46)	36.43 (9.05)	*t*(522) = −0.442, *p* = 0.659, Cohen’s *d* = −0.047
Primary diagnosis (according to ICD-10)			Chi^2^(8) = 23.661, *p* = 0.002, Cramer-*V* = 0.212
Mental and behavioral disorders due to the use of			
... alcohol (F10)	22 (20.0%)	70 (16.8%)	
... opioids (F11)	9 (8.2%)	25 (6.0%)	
... cannabinoids (F12)	25 (22.7%) °	51 (12.2%)	
... sedatives or hypnotics (F13)	0	1 (0.2%)	
... cocaine (F14)	9 (8.2%)	57 (13.7%)	
... other stimulants (F15)	10 (9.1%)	17 (4.1%)	
... multiple drug use (F19)	34 (30.9%) ^	194 (46.5%)	
Schizophrenia (F20)	0	2 (0.5)	
Reaction to severe stress, and adjustment disorders (F43)	1 (0.9%)	0	
Comorbid personality disorder	37 (34%)	67 (16%)	Chi^2^(1) = 16.962, *p* < 0.001, Cramer-*V* = 0.179
On substitution treatment	9 (8%)	11 (3%)	Chi^2^(1) = 7.327, *p* = 0.012, Cramer-*V* = 0.118
Number of prior convictions (according to criminal record extract)	6.06 (4.85)	7.05 (5.74)	Z = −1.313, *p* = 0.189, Cohen’s *d* = 0.115
Age at first conviction (in years)	23.60 (9.53)	23.04 (9.26)	*t* (523) = 0.562, *p* = 0.574, Cohen’s *d* = 0.061
Violent index offense or history of violent offenses	58 (53%)	245 (59%)	Chi^2^(1) = 1.293, *p* = 0.279, Cramer-*V* = 0.050
Duration of hospitalization in forensic psychiatric care (in months)	22.26 (6.41)	17.64 (5.52)	*t* (524) = 7.536, *p* < 0.001, Cohen’s *d* = 0.396
Time since leave (in days)	146.85 (86.56)	133.08 (111.45)	*t* (159.702) = 0.993, *p* = 0.369, Cohen’s *d* = 0.132

Note. FET = Fisher’s exact test. ° Adjusted residual ≥ 2. ^ Adjusted residual ≤ −2.

**Table 2:** Number of patients in the district hospitals of Guenzburg and Kaufbeuren who tested negative or positive for synthetic cannabinoids.

	Guenzburg *n* (%)	Kaufbeuren *n* (%)	Total
Tested negative	53 (77.9%)	155 (96.3%)	208
Tested positive	15 (22.1%)	6 (3.7%)	21
Total	68	161	

**Table 3:** Results of the binary logistic regression predicting a positive test result for synthetic cannabinoids based on site of assessment (Kaufbeuren = 0, Guenzburg = 1) and duration from leave to discharge (in days).

Predictors	Regression Coefficient	*p*	Odds-Ratio	95% Confidence Interval	
				Lower Bound	Upper Bound
Hospital	1.967	<0.001	7.147	2.628	19.437
Time since leave (in days)	0	0.962	1.000	0.995	1.005

**Table 4:** Relapses by substance class (multiple responses possible).

	Tested Positive *n* (%)
JWH	2 (5%)
A	5 (14%)
B	0
C	2 (5%)
E	1 (3%)
F	4 (11%)
H	11 (30%)
M	12 (32%)
N	0
P	0
S	0
T	0
U	0
XLR	0

**Table 5:** Relapses involving synthetic cannabinoids over time.

	Tested Negative *n* (%)	Tested Positive *n* (%)	Total
2015	0	1	1
2016	7	0	7
2017	5	1	6
2018	13	2	15
2019	32	7	39
2020	19	2	21
2021	41	2	43
2022	32	0	32
2023	37	3	40
2024	20	3	23
2025	2	0	2
Total	208	21	229

**Table 6:** Sociodemographic, clinical, and forensic characteristics of the sample, separately for patients who tested negative and positive for synthetic cannabinoids.

	Tested Negative (*n* = 208) *M* (*SD*)/*n* (%)	Tested Positive (*n* = 21) *M* (*SD*)/*n* (%)	Statistics
Sex			Chi^2^(1) = 0.101, *p* = 1.000, Cramer-*V* = 0.021
male	207 (99.5%)	21 (100%)	
female	1 (0.5%)	0	
Age at the beginning of leave (in years)	35.17 (8.41)	34.19 (5.78)	*t* (225) = 0.518, *p* = 0.605, Cohen’s *d* = 0.119
Primary diagnosis (according to ICD-10)			Chi^2^(7) = 7.136, *p* = 0.357, Cramer-*V* = 0.177
Mental and behavioral disorders due to the use of			
... alcohol (F10)	26 (12.5%)	4 (19.0%)	
... opioids (F11)	11 (5.3%)	0	
... cannabinoids (F12)	35 (16.8%)	3 (14.3%)	
... cocaine (F14)	26 (12.5%)	0	
... other stimulants (F15)	10 (4.8%)	0	
... multiple drug use (F19)	98 (47.1%)	14 (66.7%)	
Schizophrenia (F20)	1 (0.5%)	0	
Reaction to severe stress, and adjustment disorders (F43)	1 (0.5%)	0	
Comorbid personality disorder	53 (26%)	4 (19%)	Chi^2^(1) = 0.422, *p* = 0.363, Cramer-*V* = 0.043
On substitution treatment	10 (5%)	4 (19%)	Chi^2^(1) = 6.739, *p* = 0.029, Cramer-*V* = 0.172
Number of prior convictions (according to criminal record extract)	6.38 (5.17))	8.43 (5.03)	Z = −1.862, *p* = 0.063, Cohen’s *d* = 0.248
Age at first conviction (in years)	22.39 (9.06)	19.33 (4.87)	*t*(36.136) = 2.471, *p* =0.018, Cohen’s *d* = 0.349
Violent index offense or history of violent offenses	111 (53%)	18 (86%)	Chi^2^(1) = 8.114, *p* = 0.003, Cramer-*V* = 0.188
Duration of hospitalization in forensic psychiatric care (in months)	19.06 (6.16)	19.07 (6.57)	*t*(227) = −0.010, *p* = 0.992, Cohen’s *d* = 0.002
Time since leave (in days)	135.79 (107.03)	142.29 (81.04)	*t*(216) = −0.270, *p* = 0.787, Cohen’s *d* = −0.062

**Table 7:** Binary logistic regression predicting positive synthetic cannabinoid tests from substitution treatment, age at first conviction, violent offense, and time since leave.

Predictors	Regression Coefficient	*p*	Odds-Ratio	95% Confidence Interval	
				Lower Bound	Upper Bound
On substitution treatment	1.244	0.067	3.468	0.916	13.138
Age at first conviction (in years)	−0.018	0.650	0.982	0.910	1.061
Violent index offense or history of violent offenses	1.397	0.038	4.043	1.083	15.086
Time since leave (in days)	0.001	0.007	1.001	0.997	1.005

## Data Availability

The original contributions presented in this study are included in the article. Further inquiries can be directed to the corresponding author.
